# Characteristics of patients diagnosed with pancreatic cancer who access palliative care: An observational study

**DOI:** 10.1007/s11136-023-03425-x

**Published:** 2023-05-03

**Authors:** Nadia N. Khan, Sue M. Evans, Liane J. Ioannou, Charles H. C. Pilgrim, Megan Blanchard, Barbara Daveson, Jennifer Philip, John R. Zalcberg, Luc te Marvelde

**Affiliations:** 1 Cancer Research Program, School of Public Health and Preventive Medicine, Monash University, 553 St Kilda Road, Melbourne, VIC 3004 Australia; 2 Victorian Cancer Registry, Cancer Council Victoria, 615 St Kilda Road, Melbourne, VIC 3004 Australia; 3 Palliative Care Outcomes Collaboration, University of Wollongong, Northfields Avenue, Wollongong, NSW 2522 Australia; 4 Department of Medicine, University of Melbourne, St Vincent’s Hospital Campus, Victoria Pde, Fitzroy, VIC 3000 Australia

**Keywords:** Palliative care, Pancreatic cancer, Symptom distress, Problem severity

## Abstract

**Purpose:**

Despite the benefits of palliative care (PC) in pancreatic cancer, little is known about patients who access PC. This observational study examines the characteristics of patients with pancreatic cancer at their first episode of PC.

**Methods:**

First-time, specialist PC episodes captured through the Palliative Care Outcomes Collaboration (PCOC), in Victoria, Australia between 2014 and 2020, for pancreatic cancer, were identified. Multivariable logistic regression analyses examined the impact of patient- and service-level characteristics on symptom burden (measured through patient-reported outcome measures and clinician-rated scores) at first PC episode.

**Results:**

Of 2890 eligible episodes, 45% began when the patient was deteriorating and 32% ended in death. High fatigue and appetite-related distress were most common. Generally, increasing age, higher performance status and more recent year of diagnosis predicted lower symptom burden. No significant differences were noted between symptom burden of regional/remote versus major city dwellers; however, only 11% of episodes recorded the patient as a regional/remote resident. A greater proportion of first episodes for non-English-speaking patients began when the patient was unstable, deteriorating or terminal, ended in death and were more likely to be associated with high family/carer problems. Community PC setting predicted high symptom burden, with the exception of pain.

**Conclusion:**

A large proportion of first-time specialist PC episodes in pancreatic cancer begin at a deteriorating phase and end in death, suggesting late access to PC. Timely referrals to community-based specialist PC, access in regional/remote areas, as well as development of culturally diverse support systems require further investigation.

**Supplementary Information:**

The online version contains supplementary material available at 10.1007/s11136-023-03425-x.

## Plain English Summary

Palliative care can help patients with pancreatic cancer manage their symptoms and improve their quality of life. Although palliative care should be available to all patients with pancreatic cancer, we don’t know whether patients are getting this support early enough. This study aimed to understand whether certain groups of patients with pancreatic cancer are more or less likely to present to palliative care with high symptom distress or problems. By understanding which groups of patients present to palliative care with high symptom distress or problems, we can design strategies to help these patients get support earlier. We found that the majority of patients with pancreatic cancer first saw palliative care when their overall health had worsened. Only a small number of patients who saw a palliative care service included in this study lived in a regional or remote area. Patients who presented to community palliative care generally had high symptom distress and problems. Compared to English-speaking patients, a greater proportion of patients who did not speak English were deceased at the end of their first presentation and their families/carers were more likely to present with high distress. These findings indicate that patients with pancreatic cancer may not be accessing palliative care early enough. It also inspires more research on ways to make palliative care easier to access in the community and for patients who live in regional and remote areas and do not speak English.

## Introduction

The majority of patients with pancreatic cancer are diagnosed when the cancer has metastasised [1]. Coupled with a poor prognosis, patients with pancreatic cancer experience a high symptom burden [2–4], which, if inadequately managed, contributes to a poor quality of life (QoL) [5] as well as poorer survival outcomes [6]. The World Health Organisation defines palliative care as “*an approach that improves the quality of life of patients and their families faced with a life-threatening illness, through the prevention and relief of suffering by means of early identification and impeccable assessment and treatment of pain and other problems, physical, psychosocial and spiritual”* [7]. Clinical management pathways for pancreatic cancer recommend that all patients be offered a referral for PC assessment [8] and that early PC occur concurrently with active cancer treatment [9].

Engagement with PC services has been demonstrated to have numerous benefits. A systematic review and meta-analysis of 43 randomised controlled trials exploring the impact of PC on patient outcomes found PC to be associated with statistically and clinically significant improvements in QoL and symptom burden as well as reduced healthcare utilisation [10]. In pancreatic cancer specifically, early referral to PC has been associated with reduced use of futile and aggressive anti-cancer treatment towards the end of life [11], fewer acute hospital admissions and reduced healthcare costs [12]. Additionally, PC interventions have been associated with reduced depression and stress in carers of patients with advanced cancer [13].

According to an international consensus recommendation, referrals to specialist PC should be made within 3 months of advanced cancer diagnosis for patients with a median survival of one year or less [14]. Consequently, in line with this recommendation, all non-resectable patients with pancreatic cancer should ideally be referred to PC. However, a cross-sectional study conducted by Beesley et al. in a cohort of patients with pancreatic and ampullary cancers found that only 59% of non-resectable and 27% of resectable patients accessed PC services [15].

To date, there are limited studies which have explored characteristics associated with use of PC specifically in pancreatic cancer and those that have been reported are largely based on the American, Medicare-enrolled population [16]. Studies which have investigated PC use in the Australian context have tended to do so through the limited lens of in-patient PC admissions, chemotherapy in the last 30 days of life and place of death [17]. Consequently, this study aims to describe the characteristics of patients with pancreatic cancer who present to a specialist PC service in Victoria, Australia, using a comprehensive PC admissions dataset, to inform our understanding and guide potential opportunities to promote equitable access to PC. Given the variability in the cancer care management approaches across Australian States and Territories (for example, New South Wales employs a centralised model of care for patients with pancreatic cancer), this study focussed only on Victoria as a case study. Specifically, this study aims to understand the association between patient characteristics and symptom burden at initial presentation to a Victorian specialist PC service, as well as compare the demographic characteristics of patients referred to PC to the overall Victorian population of patients with pancreatic cancer.

## Methods

### Study design and data source

This retrospective cohort study used de-identified data from the Palliative Care Outcomes Collaboration (PCOC). PCOC is a voluntary national programme that measures and benchmarks patient outcomes in PC. Approximately 90% of all in-patient and community, specialist PC services across Australia are captured in PCOC [18].

To compare the characteristics of the PCOC Victorian pancreatic cancer cohort to the overall Victorian pancreatic cancer population, aggregate-level data for our population of interest, diagnosed between 2014 and 2020, were sourced from the Victorian Cancer Registry (VCR). The VCR is a state-wide, population-based registry, which captures all reportable cancer cases diagnosed across Victoria, Australia.

### Study participants and inclusion criteria

PCOC collects data for each episode of PC, with one episode being defined as a continuous period of care for a patient in one setting (i.e. hospital, private residence and residential aged care facility). A patient receiving PC is likely to have more than one episode. All episodes of PC for patients diagnosed with pancreatic cancer, who were at least 18 years of age and occurred at a Victorian service participating in PCOC, between 01 January 2014 and 31 December 2020, were identified. As we were interested in understanding the characteristics of patients using specialist PC at initial presentation to PC, only the first episode of PC recorded for each patient was included. Additionally, a single patient may have more than one ‘first episode’ of PC if they migrate between different health services. In order to mitigate this issue, first episodes which had another PC service as a referral source were excluded.

In order to understand whether there are variations in the symptom burden of patients with pancreatic cancer compared to those diagnosed with other cancers, the symptom distress scores at first episode of PC were compared between patients with pancreatic cancer and those with a 5-year relative survival lower than 50% (‘lower survival cancers’) or greater than or equal to 50% (‘higher survival cancers’), as determined by statistics reported by the Australian Institute of Health and Welfare [1]. ‘Lower survival cancers’ included cancers of the central nervous system, lung and other gastrointestinal cancers. ‘Higher survival cancers’ (across all stages) included cancers of the bone and soft tissue, breast, colorectal, haematological, head and neck, prostate, other urological, gynaecological and skin cancers. Consequently, episodes of PC for patients with a diagnosis of one of the aforementioned cancers, who met the eligibility criteria applied for pancreatic cancer (mentioned previously) were sought through PCOC.

### Study measures

The key objective of this study was to understand the association between patient characteristics and symptom burden (primary outcome) at initial presentation to PC.

Symptom distress was measured through scores obtained on each item of the PCOC Symptom Assessment Scale (SAS) [19] and symptom severity was measured through the Palliative Care Problem Severity Score (PCPSS) [20]. The PCOC SAS is a patient-rated symptom distress score rated along a scale of 0 (absent) to 10 (worst possible), with items capturing distress from pain, fatigue, nausea, appetite, difficulty sleeping, breathing, bowel functions and an ‘other’ item. The PCPSS is a clinician-rated score, with the severity of psychological/spiritual, pain, other physical symptoms (other than pain) and family/carer problem domains categorised as absent, mild, moderate or severe. Together, the PCOC SAS and PCPSS provide an indication of symptom burden. PCOC SAS and PCPSS scores measured at the start of the first phase of the first episode of PC were included in the analyses. Outcomes pertaining to pain, fatigue, appetite, psychological problems and the severity of family/carer symptoms are discussed in the main body of this paper with findings pertaining to nausea, bowel functions, breathing and difficulty sleeping included as supplemental material.

As secondary outcomes, we characterised the PCOC pancreatic cancer cohort in the context of the overall Victorian pancreatic cancer population, assessed timeliness of care (measured as median (interquartile range (IQR)) time from referral to initial contact with PC) and compared symptom burden at initial presentation to PC between pancreatic and other cancers. In order to better understand timely access, we explored the association between patient characteristics and phase type at initial presentation: stable, unstable, deteriorating and terminal. In the stable phase, a patient’s symptoms and problems are adequately controlled through an established care plan, with a plan for further interventions, and family/carer issues are also stable; when unstable, there may be a new, unanticipated problem, rapid increase in the severity of an existing problem and/or changes in family/carer circumstances; when deteriorating, the overall functional status of the patient declines, existing problems gradually worsen, a new but anticipated problem emerges and/or family/carers experience worsening distress and when terminal, death is imminent within days [21].

Independent variables included patient characteristics: sex (male or female), age group (< 55, 55–64, 65–74, 75–84 and ≥ 85), country of birth (Australia or other), preferred language (English or other), location of residence based on postcode (major city or regional/remote), year of first episode and Australian Karnofsky Modified Performance Scale Score (AKPS) measured at the beginning of the first episode of care. Decreasing AKPS score suggests poorer performance status [22]. Additionally, service setting, defined as either ‘community’ (including community not further specified, private residence, residential aged care facility and hospital ambulatory) or ‘in-patient’ (including overnight admitted and same day admitted) PC was an independent variable.

### Analyses

Univariable and multivariable logistic regression analyses explored the association between patient characteristics and symptom-related distress at initial presentation to PC. Based on existing literature [3, 23], binary outcomes for the PCOC SAS were based on a cut-off of 4, whereby scores below 4 indicated absent or mild symptoms (referred to as ‘low severity’) and scores ≥ 4 indicated moderate or severe symptoms (referred to as ‘high severity’). Binary outcomes for the PCPSS were grouped as absent/mild for ‘low severity’ and moderate/severe for ‘high severity’.

Separate multivariable models were built for each symptom or problem captured through the PCOC SAS and PCPSS. Backward stepwise regression was undertaken to identify patient characteristics significantly associated with PCOC SAS and PCPSS scores. Statistical significance was set at *p* < 0.05. Each variable that did not reach statistical significance was added to the final model separately to estimate its effect size and significance. Odds ratios (OR) and 95% confidence intervals (CIs) resulting from the final model have been reported. Comparison between VCR and PCOC cohorts were conducted using chi-square tests. All analyses were conducted using R software.

This study was approved by the Monash University Human Research Ethics Committee (project: 2020–26021-49509).

## Results

Between 2014 and 2020, 2890 eligible first episodes of specialist PC were identified. The majority of episodes had patients recorded as being male (52.5%), born in a country other than Australia (53.1%), English speaking (84.4%), residing in major cities (89.2%) and aged ≥ 65 years (77.5%). Public hospitals were the predominant referral source (50.5%) (Table 1).

**Table 1:** Characteristics of pancreatic cancer cases at first episode of PC registered in the Palliative Care Outcomes Collaboration dataset between 2014 and 2020

		Initial presentation cohort^**#**^	Deceased at the end of initial presentation (i.e. first episode of palliative care)	p-value*
		n (%)		
Number of first episodes of PC		2890	933 (32.0)	n/a
Year of first episode^	2014	337 (11.7)	118 (35.0)	0.82
	2015	340 (11.7)	108 (31.8)	
	2016	430 (14.9)	134 (31.2)	
	2017	484 (16.7)	165 (34.1)	
	2018	429 (14.8)	139 (32.4)	
	2019	401 (13.9)	123 (30.7)	
	2020	469 (16.2)	146 (31.1)	
Sex	Male	1516 (52.5)	472 (31.1)	0.18
	Female	1374 (47.5)	461 (33.6)	
Country of Birth	Australia	1355 (46.9)	446 (32.9)	0.52
	Other	1535 (53.1)	487 (31.7)	
Preferred Language	English	2440 (84.4)	762 (31.2)	0.01
	Non-English	450 (15.6)	171 (38.0)	
Location of residence^?^	Major city	2579 (89.2)	850 (33.0)	0.03
	Regional/Remote	311 (10.8)	83 (26.7)	
Age group	< 55	192 (6.64)	47 (24.5)	< 0.01
	55–64	456 (15.8)	131 (28.7)	
	65–74	773 (26.7)	244 (31.6)	
	75–84	952 (32.9)	307 (32.2)	
	85 +	517 (17.9)	204 (39.5)	
Phase type at first episode	Stable	984 (34.0)	165 (16.8)	< 0.01
	Unstable	505 (17.5)	221 (43.8)	
	Deteriorating	1302 (45.1)	458 (35.2)	
	Terminal	99 (3.43)	89 (89.9)	
Service type	Community	1814 (62.8)	377 (20.8)	< 0.01
	In-patient	1076 (37.2)	556 (51.7)	
Referral Source	Public Hospital	1460 (50.5)	n/a	n/a
	Private Hospital	258 (8.9)	n/a	
	Emergency Department (Public or Private)	97 (3.4)	n/a	
	Community Services	823 (28.5)	n/a	
	Other	252 (8.7)	n/a	

^*#*^*Includes cases who were registered as having an episode identifier of ‘1’ and were referred to their first episode from a non-PC service*

^*^p-value calculated using Chi-Square Test of Independence

^*^*^*Episode refers to a continuous period of care for a patient in one setting (i.e. hospital, private residence and residential aged care facility*). An episode begins the day the patient is assessed by the palliative care provider and there is agreement between the patient and the service that the patient is ready to receive palliative care

*?Location of residence classification based on Australian Statistical Geography Standard (ASGS) 2016 Remoteness Structure. ‘Regional/Remote’ includes Inner Regional, Outer Regional, Remote and Very Remote Australia. PCOC’s remoteness structure is based on the postcode of the usual residence of the patient*

### Phase type

Over 45% of first-time PC episodes began when the patient was in a deteriorating phase (Table 1). A greater proportion of PC episodes which began when the patient was unstable, deteriorating or terminal was reported in patients with a preference for a language other than English, compared to episodes involving English-speaking patients (72% vs 65%, respectively) (Table S1).

### Episode length and discharge mode

The median time from referral to initial contact with PC was 3 (IQR: 0–8) days. On average, first episodes lasted for a median of 13 (IQR: 4–41) days. The majority of episodes either ended in death (*n* = 933, 32%) or discharge to in-patient PC (*n* = 898, 31%). Of the episodes which ended in death, the median (IQR) length of these episodes was 10 (IQR: 4–27) days. Most notably, a greater proportion of deaths were observed for episodes for patients who preferred a language other than English (38% versus 31.2% in English-speaking patients, *p* = 0.01) and those who resided in a major city (33% versus 26.7% in regional/remote residents, *p* = 0.03) (Table 1).

### Symptom burden at presentation

Results of multivariable logistic regression analyses for predictors of high distress from pain, fatigue, appetite and psychological/spiritual and family/carer problems at first episode of specialist PC are discussed below, with univariable logistic regression outputs included as supplemental material (Tables S2 and S3). A breakdown of symptom distress by patient- and service-level characteristics is provided as supplemental material (Table S4).

### Pain

Two measurements of pain collected at initial presentation were analysed, one patient-rated (measured through the PCOC SAS) and the other clinician-rated (measured through the PCPSS).

Approximately 19% (*n* = 562) of first episodes of PC commenced with high patient distress related to pain (≥ 4 on PCOC SAS). Episodes for patients who were aged 55 years or above were less likely to be associated with high self-rated distress related to pain, compared to episodes for those aged < 55 years. The likelihood of presenting with high distress related to pain also decreased over time. In-patient PC episodes were more likely to be associated with high distress related to pain compared to community PC episodes (Table 2). Similar proportions and trends were observed for pain measured along the PCPSS (Table 3).

**Table 2:** Multivariable logistic regression analysis of predictors of high Palliative Care Outcomes Collaboration (PCOC) Symptom Assessment Scale (SAS)* score in pancreatic cancer at initial presentation to specialist palliative care^#^

Demographic characteristics		High distress related to pain *(n* = *562 (19.4%))*			High distress related to fatigue *(n* = *938 (32.5%))*			High distress related to appetite *(n* = *599 (20.7%))*		
		aOR	95% CI	*p* value	aOR	95% CI	*p* value	aOR	95% CI	*p* value
Sex	Male	1	Reference	0.95	1	Reference	0.20	1	Reference	0.57
	Female	1.01	0.83–1.22		1.11	0.94–1.32		1.05	0.88–1.27	
Country of Birth	Australia	1	Ref	0.84	1	Reference	< 0.001	1	Reference	0.003
	Other	0.98	0.81–1.19		0.72	0.60–0.86		0.76	0.63–0.91	
Preferred Language	English	1	Ref	0.42	1	Reference	0.04	1	Reference	0.08
	Non-English	0.89	0.67–1.18		0.75	0.57–0.98		0.76	0.56–1.03	
Location of residence^?^	Major city	1	Reference	0.28	1	Reference	0.33	1	Reference	0.80
	Regional/Remote	1.19	0.87–1.61		0.87	0.66–1.15		0.96	0.70–1.30	
Age group	< 55	1	Reference	< 0.001	1	Reference	0.77	1	Reference	0.47
	55–64	0.53	0.36–0.77		1.03	0.70–1.51		1.13	0.73–1.77	
	65–74	0.39	0.27–0.56		0.91	0.63–1.30		1.28	0.86–1.97	
	75–84	0.35	0.25–0.50		0.88	0.62–1.26		1.37	0.92–2.09	
	≥ 85	0.23	0.15–0.35		0.88	0.60–1.30		1.26	0.82–1.97	
Episode Start Year	–	0.89	0.84–0.93	< 0.001	0.77	0.73–0.80	< 0.001	0.78	0.74–0.82	< 0.001
AKPS at start of episode	–	0.99	0.99–1.00	0.03	0.98	0.97–0.98	< 0.001	0.99	0.99–1.00	0.08
Service setting	Community	1	Reference		1	Reference		1	Reference	
	In-patient	1.73	1.39–2.14	< 0.001	0.56	0.46–0.68	< 0.001	0.90	0.74–1.10	0.30

aOR adjusted odds ratio, CI Confidence Interval, AKPS Australia-modified Karnofsky Performance Status

^#^All analyses were adjusted for sex, country of birth, preferred language, location of residence, age group, episode start year, service setting and AKPS score measured at the start of the first episode of palliative care

*High pain, appetite distress and fatigue defined as a PCOC SAS score of ≥ 4 (i.e. low pain, appetite distress, fatigue defined as a PCOC SAS score < 4)

? Location of residence classification based on Australian Statistical Geography Standard (ASGS) 2016 Remoteness Structure. ‘Regional/Remote’ includes Inner Regional, Outer Regional, Remote and Very Remote Australia. PCOC’s remoteness structure is based on the postcode of the usual residence of the patient

**Table 3:** Multivariable logistic regression analysis of predictors of high Palliative Care Problem Severity (PCPSS)* score in pancreatic cancer at initial presentation to specialist palliative care^#^

Demographic characteristics		High severity of psychological/spiritual problems *(n* = *373 (12.9%))*			High severity of pain *(n* = *543 (18.8%))*			High severity of family/carer problems *(n* = *541 (18.7%))*		
		aOR	95% CI	p value	aOR	95% CI	p value	aOR	95% CI	p value
Sex	Male	1	Reference	0.24	1	Reference	0.78	1	Reference	0.02
	Female	1.14	0.91–1.43		0.97	0.80–1.18		**0.78**	0.64–0.95	
Country of Birth	Australia	1	Reference	0.16		Reference	0.93	1	Reference	0.93
	Other	0.85	0.68–1.06		0.99	0.82–1.20		1.01	0.81–1.26	
Preferred Language	English	1	Reference	0.13	1	Reference	0.92	1	Reference	0.04
	Non-English	0.77	0.55–1.08		1.01	0.77–1.32		**1.32**	1.01–1.72	
Location of residence^?^	Major city	1	Reference	0.44	1	Reference	0.09	1	Reference	0.60
	Regional/Remote	0.86	0.58–1.24		1.30	0.96–1.74		0.91	0.64–1.28	
Age group	< 55	1	Reference	< 0.001	1	Reference	< 0.001	1	Reference	< 0.001
	55–64	**0.83**	0.53–1.32		**0.62**	0.43–0.91		**0.83**	0.54–1.30	
	65–74	**0.74**	0.49–1.15		**0.44**	0.31–0.63		**0.86**	0.58–1.31	
	75–84	**0.54**	0.36–0.84		**0.37**	0.26–0.52		**0.75**	0.51–1.13	
	85 +	**0.38**	0.23–0.62		**0.23**	0.15–0.35		**0.43**	0.28–0.69	
Episode Start Year	-	**0.83**	0.79–0.88	< 0.001	**0.94**	0.89–0.99	0.01	**0.82**	0.77–0.86	< 0.001
AKPS at start of episode	-	**0.99**	0.98–0.99	< 0.001	**0.99**	0.98–1.00	0.01	**0.97**	0.96–0.98	< 0.001
Service setting	Community	1	Reference		1	Reference		1	Reference	
	In-patient	**0.75**	0.58–0.97	0.03	**1.36**	1.10–1.69	0.005	**0.44**	0.34–0.55	< 0.001

aOR, adjusted odds ratio; CI, Confidence Interval; AKPS, Australia-modified Karnofsky Performance Status

*High severity of psychological/spiritual problems, pain and family/carer problems defined as a PCPSS of moderate or severe (i.e. low severity of problems defined as a PCPSS of absent or mild)

^#^All analyses were adjusted for sex, country of birth, preferred language, location of residence, age group, episode start year, service setting and AKPS score measured at the start of the first episode of palliative care

? Location of residence classification based on Australian Statistical Geography Standard (ASGS) 2016 Remoteness Structure. ‘Regional/Remote’ includes Inner Regional, Outer Regional, Remote and Very Remote Australia. PCOC’s remoteness structure is based on the postcode of the usual residence of the patient

### Fatigue

High distress related to fatigue (≥ 4 on PCOC SAS) was recorded for 32.5% (*n* = 938) of first episodes of PC. Episodes for patients who were born in a country other than Australia, preferred a language other than English, accessed PC more recently, had higher AKPS scores and presented to an in-patient PC service were less likely to be associated with high distress related to fatigue compared to their counterparts (Table 2).

### Appetite

High distress related to appetite (≥ 4 on PCOC SAS) was recorded for 20.7% (*n* = 599) of first episodes of PC. Episodes for patients who were born in a country other than Australia and those who accessed PC more recently, were less likely to be associated with high distress related to appetite compared to their counterparts (Table 2).

### Psychological/spiritual problems

High psychological/spiritual problems (moderate/severe on PCPSS) was recorded for 12.9% (*n* = 373) of first episodes of PC. Episodes of PC for patients aged above 55 years, those who accessed PC more recently, had higher AKPS score and presented to an in-patient PC service were less likely to be associated with moderate/severe psychological/spiritual distress compared to their counterparts (Table 3).

### Family/carer problems

High levels of family/carer problems (moderate/severe on PCPSS) were recorded for 18.7% (*n* = 541) of first episodes of PC. Episodes for patients who preferred a language other than English were more likely to be associated with high family/carer problems compared to episodes for patients who spoke English. Episodes for female patients and those aged at least 55 years were less likely to be associated with high family/carer problems. Additionally, episodes which occurred more recently, at an in-patient setting or for patients with increasing AKPS scores were less likely to be associated with high family/carer problems (Table 3).

### Comparison between PCOC and VCR

A total of 6587 newly diagnosed cases with pancreatic cancer were registered in the VCR dataset between 2014 and 2020. During this time, 2890 first-time episodes of PC in pancreatic cancer were identified through PCOC. When comparing the two populations, there were significant differences between country of birth, location of residence and age group, with the most notable being that a smaller proportion of regional/remote area residents were registered as receiving specialist PC in PCOC (27.2% of VCR cases reside in regional/remote areas  v. 10.8% of PCOC cases). No significant difference was noted between the two populations when comparing sex (Table 4).

**Table 4:** Comparison of demographic characteristics of patients with pancreatic cancer registered in the Victorian Cancer Registry (VCR) versus first episodes of specialist palliative care registered in the Palliative Care Outcomes Collaboration (PCOC) between 2014 and 2020

Variable		Victorian Cancer Registry (2014–2020) n (%)	Palliative Care Outcomes Collaboration (2014–2020) n (%)	P value
		6587 (100.0)	2890 (100.0)	
Tumour Morphology	PDAC^^^	5764 (87.5)	n/a	–
	Non-PDAC	823 (12.5)	n/a	
Sex	Female	3211 (48.7)	1374 (47.5)	0.29
	Male	3376 (51.3)	1516 (52.5)	
Country of Birth	Australia	3775 (57.3)	1355 (46.9)	< 0.001
	Other	2668 (40.5)	1535 (53.1)	
	Unknown	144 (2.2)	0 (0.0)	
Location of residence^?^	Major cities	4785 (72.7)	2579 (89.0)	< 0.001
	Regional/Remote	1794 (27.2)	311 (10.8)	
	Missing	8 (0.12)	0 (0.0)	
Age Group^#^	< 55	627 (9.5)	192 (6.6)	< 0.001
	55–64	1068 (16.2)	456 (15.8)	
	65–74	1877 (28.5)	773 (26.7)	
	75–84	1847 (28.0)	952 (32.9)	
	≥ 85	1168 (17.7)	517 (17.9)	

PDAC Pancreatic Ductal Adenocarcinoma

^^^PDAC tumours defined as those with morphology codes 8000 (neoplasm, malignant), 8010 (carcinoma, NOS), 8140 (adenocarcinoma, NOS) and 8500 (infiltrating duct carcinoma, NOS)

^#^Age group for VCR refers to age at diagnosis. Age group for PCOC refers to age at initial presentation to palliative care

^?^Location of residence classification based on Australian Statistical Geography Standard (ASGS) 2016 Remoteness Structure. ‘Regional/Remote’ includes Inner Regional, Outer Regional, Remote and Very Remote Australia. PCOC’s remoteness structure is based on the postcode of the usual residence of the patient. VCR’s remoteness structure is based on statistical area level 1 of the usual residence of the patient

### Symptom burden in pancreatic cancer versus lower and higher survival cancers

Pain, fatigue, appetite distress, psychological/spiritual problems and family/carer problems were compared between first episodes of PC for patients with pancreatic versus lower and higher survival cancers, respectively (refer to Table S5 for demographic characteristics of lower and higher survival cancer patients registered in PCOC). First episodes of PC for patients with pancreatic cancer were more likely to be associated with fatigue and appetite distress compared to episodes for lower as well as higher survival cancer patients (Tables S6 and S7). A greater likelihood of high pain in pancreatic cancer at first PC episode was observed when comparing against lower survival cancers (for both PCOC SAS and PCPSS pain scores) and higher survival cancers (for PCOC SAS only) (Table S7). No significant differences were noted when analysing psychological/spiritual problems, nor family/carer problem scores.

## Discussion

This study presents one of the largest series of symptom and problem profiling in patients with pancreatic cancer at presentation to specialist PC. Our findings indicate that the likelihood of experiencing high symptom distress at the commencement of first PC episode decreased over time, perhaps suggesting that timing of access to PC has improved over time. This is also coupled with the finding that general access to PC has increased over time. However, given the majority of first PC episodes began at a deteriorating phase, coupled with the fact that 32% of episodes ended in death, this may suggest that access is not occurring early enough. Aside from symptom relief, early referrals to PC also offer the opportunity for early discussion of goals and setting plans which may mitigate escalation of care at the end of life. Consequently, implementation of early PC in routine practice, as being explored by the Care Plus trial [24], may be highly beneficial for both improving patient outcomes and reducing acute hospitalisations.

Compared to the state-wide pancreatic cancer population, a significantly smaller proportion of the PCOC cohort were registered as residing in a regional or rural area of Victoria. This may be explained by the fact that regional and rural areas often have different models of PC (for example, each health region/district may not have a dedicated specialist PC team and may instead rely on a range of staff who work across multiple services). These services are not defined as ‘specialist’ PC care (and as such, are not included in the PCOC dataset). Surprisingly, symptom distress at initial presentation to PC for patients residing in regional and rural areas was comparable to those residing in metropolitan regions. However, given the limited capture of this sub-population, it is important to further interrogate symptom distress more broadly across all patients with pancreatic cancer who reside in regional and rural areas, particularly for those who access generalist supportive care only. Equitable access to high-quality specialist PC in regional areas may be mediated through a hybrid model of care that includes both in-person and telehealth consultations, with trained specialist PC providers [25].

High distress from fatigue was the most commonly experienced issue, affecting more than 30% of our cohort, followed by appetite distress (affecting 21% of the cohort). These findings are unsurprising as previous studies have indicated that fatigue- as well as appetite-related distress are commonly experienced issues in the pancreatic cancer population [26]. Additionally, our findings also mirror previous studies in that pancreatic cancer patients were more likely to experience high fatigue- and appetite-related issues compared to patients with other lower and higher survival cancers [27]. Facilitation of early access to multidisciplinary supportive care, particularly to dietetic support, is highly desired by patients with pancreatic cancer [28] and should form a key priority area in the delivery of care.

Surprisingly, only ~ 20% of patients experienced high distress related to pain at presentation to PC. A recent study exploring the symptom burden of advanced pancreatic cancer patients found that the median score at baseline for pain was 1, when measured along the ESAS [3]. Our findings also mirror the distribution of pain distress scores reported by other studies using PCOC data [19]. However, literature also suggests that pain affects 80% of patients with pancreatic cancer, with nearly half requiring strong opioids for pain relief [29]. It is also possible that many patients included in our study had received pain management support prior to their first episode of PC; however, this data is not collected through PCOC. The true prevalence of distress related to pain in pancreatic cancer, along the disease trajectory, requires further interrogation so that patients can be offered appropriate support when most needed.

With the exception of pain, admission to an in-patient PC service was generally a predictor of lower symptom distress/problem severity at initial presentation, compared to community setting. A study conducted in a US-based community-dwelling population of patients with advanced cancer reported similar findings in that a significant proportion of patients reported moderate/severe symptom distress when first engaging community PC [30]. Qualitative studies involving patients and caregivers as well as community PC service providers may help to identify barriers and enablers of timely access to community-based specialist PC.

Fatigue at initial presentation was lower in pancreatic cancer patients who preferred a language other than English, with no differences observed in pain, appetite or psychological distress, despite a higher proportion presenting when unstable, deteriorating or terminal. Whilst certain cultural lifestyle factors may potentially enable better symptom management and therefore explain the reduced likelihood to experience fatigue, it is difficult to elucidate the true causation as literature regarding symptom distress in culturally and linguistically diverse (CALD) cancer patients is limited and conflicting. For example, one study found that migrants with cancer were more likely to report worse health-related QoL and higher emotional distress compared to Anglo-Saxon patients [31], whilst another found that CALD patients reported a similar level of symptom distress (measured through the Distress Thermometer and Problem Checklist) to non-CALD patients [32]. Similar to our findings, a recent study using PCOC data reported that cancer patients preferring non-English languages were less likely to report symptoms or problems, except for family/carer problems [33].

Language has been identified as a major barrier to the assessment and management of symptoms in PC [34]. System-level barriers, such as structural racism, may also hinder accurate reporting or disclosure of symptom distress [35]. One way to mitigate these issues is to ensure that Non-English-speaking patients are provided with opportunities to participate in research studies exploring health service delivery assessment [36]. Additionally, tools which are validated in languages other than English should be routinely used when required. It remains unclear whether translated versions were used with patients represented in this dataset. Finally, it is important to integrate PC into standard cancer care pathways so that it is considered at key points along the care continuum [37]. Our finding that a significantly greater proportion of patients who preferred a language other than English were deceased at the end of their first episode of PC suggests that timely access ought to be specifically explored in this population.

Family/carer problem scores were significantly higher at presentation for patients with pancreatic cancer who preferred a language other than English, compared to English-speaking patients. Interestingly, whilst this effect was observed within families/carers, it was not reflected in the psychological distress scores of patients themselves. A recent qualitative study into the supportive care needs of patients diagnosed with pancreatic and oesophagogastric cancers has reported that patients often rely on carers for emotional support and do not feel the need to seek professional support. Their carers, however, desire professional support for themselves [28]. Whilst the unmet needs of cancer carers, particularly those who are of a CALD background, have received limited attention to date, a survey conducted by Carers NSW found that carers from CALD backgrounds report higher levels of psychological distress and less social support [38]. Further investigation of the unmet needs of CALD cancer carers is required to inform, design and implement culturally appropriate services and resources to reduce the burden they may experience.

Although the odds of presenting with high pain, psychological/spiritual distress or family/carer problems decreased with age, it is important to interpret these findings with caution as the reason behind this association is not clearly understood. It has been posited that elderly patients are generally more emotionally resilient, have fewer competing demands and are more economically stable compared to younger patients [39, 40] thereby potentially explaining our findings. However, elderly patients may also under-report distress due to various factors, such as memory/hearing loss, confusion, fear of being a burden and/or stoicism [41].

To our knowledge, this is the first in-depth exploration of the characteristics and symptom burden of patients with pancreatic cancer who present to specialist PC. Our study, however, has several limitations. Despite capturing 90% of specialist PC services across Australia, PCOC is a voluntary service and therefore does not capture all PC presentations, thereby limiting the generalisability of our findings to the overall pancreatic cancer population. Additionally, given our inability to uniquely identify patients across all services, it is possible that some patients who presented to multiple different services during the eligible time period may have been counted more than once. Lack of clinical data and date of diagnosis did not allow us to look at symptom distress along the disease trajectory, timeliness of access to PC, nor survival outcomes. Socioeconomic status for patients was also not available. Future studies should interrogate the association between SES and PC use as low SES has been shown to be a predictor of poorer access to healthcare services [16]. Nevertheless, to our knowledge this is the largest series of symptoms and problem profile of people with pancreatic cancer and represents an important baseline for future studies in this uncommon cancer.

## Conclusion

Although a large proportion of first-time PC episodes in pancreatic cancer begin when the patient is deteriorating and end in death, high symptom distress (with the exception of fatigue) at presentation is relatively low and not generally associated with a patient’s demographic characteristics. However, the higher likelihood of presenting to community-based specialist PC with high symptom distress suggests earlier referral pathways require further interrogation. Access to PC ought to be explored in CALD communities given a greater proportion of deaths amongst non-English-speaking patients at initial presentation and higher family/carer problems scores were observed. Additionally, reasons for underrepresentation of patients residing in regional and remote areas who access specialist PC requires further investigation.

## Supplementary Information

Below is the link to the electronic supplementary material.

Supplementary file1 (DOCX 57 KB)

## Data Availability

The data that support the findings of this study are available from the Palliative Care Outcomes Collaboration, upon reasonable request.
