# Supplementary material for: Characteristics of patients diagnosed with pancreatic cancer who access palliative care: An observational study
Source: Qual Life Res. 2023 May 3;32(9):2617–27. doi: 10.1007/s11136-023-03425-x (PMC10393853; doi:10.1007/s11136-023-03425-x)
Supplement: Supplementary file 1 — Supplementary file1 (DOCX 57 KB) [file 11136_2023_3425_MOESM1_ESM.docx]

Table S1: Influence of demographics characteristics on phase type* at initial presentation to palliative care in patients with pancreatic cancer

|  | | **Phase type at initial presentation** | | | | |
| --- | --- | --- | --- | --- | --- | --- |
|  |  | **Stable**  **(n=984)** | **Unstable**  **(n=505)** | **Deteriorating**  **(n=1302)** | **Terminal**  **(n=99)** | **p value** |
|  | | ***n (%)*** | | | |  |
| **Sex** | **Male** | 509 (33.6) | 264 (17.4) | 677 (44.7) | 66 (4.35) | 0.04 |
|  | **Female** | 475 (34.6) | 241 (17.5) | 625 (45.5) | 33 (2.40) |  |
| **Country of Birth** | **Australia** | 463 (34.2) | 250 (18.5) | 590 (43.5) | 52 (3.84) | 0.27 |
|  | **Other** | 521 (33.9) | 255 (16.6) | 712 (46.4) | 47 (3.06) |  |
| **Preferred Language** | **English** | 858 (35.2) | 418 (17.1) | 1088 (44.6) | 76 (3.11) | 0.008 |
|  | **Non-English** | 126 (28.0) | 87 (19.3) | 214 (47.6) | 23 (5.11) |  |
| **Location of residence** | **Major city** | 855 (33.2) | 444 (17.2) | 1197 (46.4) | 83 (3.2) | <0.001 |
|  | **Regional/Remote** | 129 (41.5) | 61 (19.6) | 105 (33.8) | 16 (5.1) |  |
| **Age group** | **<55** | 47 (24.5) | 54 (28.1) | 83 (43.2) | 8 (4.2) | 0.002 |
|  | **55-64** | 147 (32.2) | 93 (20.4) | 204 (44.7) | 12 (2.6) |  |
|  | **65-74** | 269 (34.8) | 128 (16.6) | 357 (46.2) | 19 (2.5) |  |
|  | **75-84** | 329 (34.6) | 156 (16.4) | 429 (45.1) | 38 (4.0) |  |
|  | **≥85** | 192 (37.1) | 74 (14.3) | 229 (44.3) | 22 (4.3) |  |
| **Episode start year** | **2014** | 133 (39.5) | 70 (20.8) | 124 (36.8) | 10 (3.0) | <0.001 |
|  | **2015** | 101 (29.7) | 78 (22.9) | 151 (44.4) | 10 (2.9) |  |
|  | **2016** | 135 (31.9) | 89 (20.7) | 190 (44.2) | 16 (3.7) |  |
|  | **2017** | 173 (35.7) | 98 (20.2) | 198 (40.9) | 15 (3.1) |  |
|  | **2018** | 142 (33.1) | 76 (17.7) | 196 (45.7) | 15 (3.5) |  |
|  | **2019** | 150 (37.4) | 46 (11.5) | 190 (47.4) | 15 (3.7) |  |
|  | **2020** | 150 (32.0) | 48 (10.2) | 253 (53.9) | 18 (3.8) |  |
| **Service setting** | **Community** | 770 (42.4) | 149 (8.2) | 862 (47.5) | 33 (1.8) | <0.001 |
|  | **In-patient** | 214 (19.9) | 356 (33.1) | 440 (40.8) | 66 (6.1) |  |

*Note: Percentages calculated as row percentages. Refer to Table 1 for sample size of each sub-group.*

**Phase type refers to stage of the patient illness in terms of four phases: stable, unstable, deteriorating and terminal.*

Table S2.1: Univariable logistic regression analysis of predictors of high Symptom Assessment Scale (SAS)* score in pancreatic cancer at initial presentation to palliative care

|  | | **High distress related to Pain**  **(n=562)** | | | **High distress related to Appetite**  **(n=877)** | | | **High distress related to Fatigue**  **(n=1343)** | | |
| --- | --- | --- | --- | --- | --- | --- | --- | --- | --- | --- |
| **Demographic characteristics** | | **OR** | **95% CI** | **P value** | **OR** | **95% CI** | **P value** | **OR** | **95% CI** | **P value** |
| **Sex** | Male | 1 | ref | 0.45 | 1 | reference | 0.46 | 1 | reference | 0.11 |
|  | Female | 0.93 | 0.78-1.12 |  | 1.07 | 0.89-1.28 |  | 1.14 | 0.97-1.33 |  |
| **Country of Birth** | Australia | 1 | ref | 0.31 | 1 | reference | 0.002 | 1 | reference | <0.001 |
|  | Other | 0.91 | 0.75-1.09 |  | **0.75** | 0.63-0.91 |  | **0.70** | 0.59-0.82 |  |
| **Preferred Language** | English | 1 | ref | 0.45 | 1 | reference | 0.004 | 1 | reference | <0.001 |
|  | Non-English | 0.90 | 0.69-1.17 |  | **0.66** | 0.50-0.87 |  | **0.63** | 0.49-0.80 |  |
| **Location of residence** | Major city | 1 | ref | 0.43 | 1 | reference | 0.92 | 1 | reference | 0.34 |
|  | Regional/Remote | 1.13 | 0.83-1.51 |  | 0.98 | 0.73-1.32 |  | 0.88 | 0.67-1.14 |  |
| **Age group** | <55 | 1 | ref |  | 1 | reference |  | 1 | reference |  |
|  | 55-64 | **0.52** | 0.36-0.75 | <0.001 | 1.04 | 0.68-1.62 | 0.85 | 0.93 | 0.65-1.34 | 0.69 |
|  | 65-74 | **0.39** | 0.27-0.55 | <0.001 | 1.14 | 0.77-1.73 | 0.53 | 0.85 | 0.61-1.20 | 0.35 |
|  | 75-84 | **0.36** | 0.25-0.50 | <0.001 | 1.21 | 0.82-1.82 | 0.36 | 0.84 | 0.60-1.17 | 0.30 |
|  | ≥85 | **0.23** | 0.15-0.34 | <0.001 | 1.09 | 0.72-1.68 | 0.70 | 0.85 | 0.59-1.21 | 0.35 |
| **Episode Start Year** | - | **0.90** | 0.86-0.94 | <0.001 | **0.78** | 0.74-0.82 | <0.001 | **0.77** | 0.74-0.80 | <0.001 |
| **AKPS at start of episode** | - | **0.99** | 0.98-1.00 | <0.001 | 1.00 | 0.99-1.00 | 0.261 | **0.99** | 0.98-1.00 | <0.001 |
| **Service type** | Community | 1 | ref | <0.001 | 1 | reference |  | 1 | reference |  |
|  | In-patient | **1.83** | 1.52-2.21 |  | 0.85 | 0.70-1.04 | 0.11 | **0.68** | 0.57-0.80 | <0.001 |

*Abbreviations: CI, Confidence Interval; AKPS, Australia-modified Karnofsky Performance Status).*

**High pain, appetite distress and fatigue defined as a PCOC SAS score of ≥4 (i.e. low pain, appetite distress, fatigue defined as a PCOC SAS score <4)*

Table S2.2: Univariable logistic regression analysis of predictors of high Symptom Assessment Scale (SAS) score* in pancreatic cancer at initial presentation to palliative care

|  | | **High distress related to Bowels**  **(n=292, 10.1%)** | | | **High distress related to Difficulty sleeping**  **(n= 222, 7.7%)** | | | **High distress related to Breathing**  **(n= 139, 4.8%)** | | | **High distress related to Nausea**  **(n= 229, 7.9%)** | | |
| --- | --- | --- | --- | --- | --- | --- | --- | --- | --- | --- | --- | --- | --- |
| **Demographic characteristics** | | **OR** | **95% CI** | **p value** | **OR** | **95% CI** | **p value** | **OR** | **95% CI** | **p value** | **OR** | **95% CI** | **p value** |
| **Sex** | Male | 1 | reference | 0.84 | 1 | reference | 0.46 | 1 | reference | 0.96 | 1 | reference | 0.02 |
|  | Female | 1.02 | 0.80-1.31 |  | 0.90 | 0.68-1.19 |  | 1.01 | 0.72-1.42 |  | **1.39** | 1.06-1.83 |  |
| **Country of Birth** | Australia | 1 | reference | 0.003 | 1 | reference | 0.11 | 1 | reference | 0.09 | 1 | reference | 0.02 |
|  | Other | **0.69** | 0.54-0.89 |  | 0.80 | 0.61-1.05 |  | 0.75 | 0.53-1.05 |  | **0.72** | 0.55-0.94 |  |
| **Preferred Language** | English | 1 | reference | 0.30 | 1 | reference | 0.32 | 1 | reference | 0.91 | 1 | reference | 0.97 |
|  | Non-English | 0.83 | 0.57-1.17 |  | 0.81 | 0.53-1.21 |  | 0.97 | 0.58-1.55 |  | 1.01 | 0.68-1.46 |  |
| **Location of residence** | Major city | 1 | reference | 0.71 | 1 | reference | 0.13 | 1 | reference | 0.31 | 1 | reference | 0.67 |
|  | Regional/Remote | 0.93 | 0.61-1.37 |  | 1.37 | 0.90-2.04 |  | 0.72 | 0.36-1.29 |  | 1.10 | 0.70-1.66 |  |
| **Age group** | <55 | 1 | reference |  | 1 | reference |  | 1 | reference |  | 1 | reference |  |
|  | 55-64 | 0.71 | 0.42-1.22 | 0.20 | 1.35 | 0.75-2.57 | 0.33 | 1.13 | 0.51-2.75 | 0.77 | 1.18 | 0.65-2.24 | 0.61 |
|  | 65-74 | 0.69 | 0.43-1.14 | 0.14 | 0.93 | 0.52-1.74 | 0.81 | 1.22 | 0.51-2.75 | 0.62 | 1.03 | 0.59-1.92 | 0.92 |
|  | 75-84 | 0.71 | 0.45-1.17 | 0.16 | 0.83 | 0.47-1.55 | 0.54 | 1.24 | 0.61-2.88 | 0.58 | 0.91 | 0.52-1.69 | 0.76 |
|  | ≥85 | 0.82 | 0.50-1.38 | 0.45 | 0.98 | 0.54-1.87 | 0.95 | 0.96 | 0.43-2.34 | 0.92 | 1.04 | 0.57-1.98 | 0.91 |
| **Episode Start Year** | - | **0.93** | 0.87-0.99 | 0.03 | **0.88** | 0.82-0.94 | <0.001 | 0.92 | 0.84-1.00 | 0.06 | **0.90** | 084-0.97 | 0.004 |
| **AKPS at start of episode** | - | 1.00 | 0.99-1.00 | 0.39 | 1.00 | 0.99-1.01 | 0.79 | **0.97** | 0.96-0.98 | <0.001 | **0.99** | 0.98-1.00 | 0.003 |
| **Service type** | Community | 1 | reference | <0.001 | 1 | reference | 0.22 | 1 | reference | <0.001 | 1 | reference |  |
|  | In-patient | **1.91** | 1.49-2.44 |  | 1.19 | 0.90-1.58 |  | **2.23** | 1.58-3.15 |  |  |  |  |

*Abbreviations: CI, Confidence Interval; AKPS, Australia-modified Karnofsky Performance Status.*

**High distress from bowels, difficulty sleeping, breathing and nausea defined as a PCOC SAS score of ≥4 (i.e. low distress defined as a PCOC SAS score of <4)*

Table S3: Univariable logistic regression analysis of predictors of high Palliative Care Problem Severity (PCPSS) score* in pancreatic cancer at initial presentation to palliative care

|  | | **High severity of psychological/spiritual problems**  **(n= 373, 12.9%)** | | | **High severity of pain**  **(n= 543, 18.8%)** | | | **High severity of family/carer problems**  **(n= 541, 18.7%)** | | |
| --- | --- | --- | --- | --- | --- | --- | --- | --- | --- | --- |
| **Demographic characteristics** | | **OR** | **95% CI** | **p value** | **OR** | **95% CI** | **P value** | **OR** | **95% CI** | **P value** |
| **Sex** | Male | 1 | reference | 0.45 | 1 | reference | 0.26 | 1 | reference | 0.01 |
|  | Female | 1.09 | 0.87-1.35 |  | 0.90 | 0.74-1.08 |  | **0.78** | 0.64-0.94 |  |
| **Country of Birth** | Australia | 1 | reference | 0.09 | 1 | reference | 0.46 | 1 | reference | 0.32 |
|  | Other | 0.83 | 0.66-1.03 |  | 0.93 | 0.77-1.12 |  | 1.10 | 0.91-1.33 |  |
| **Preferred Language** | English | 1 | reference | 0.08 | 1 | reference | 0.90 | 1 | reference | 0.12 |
|  | Non-English | 0.75 | 0.54-1.02 |  | 0.98 | 0.76-1.27 |  | 1.23 | 0.95-1.56 |  |
| **Location of residence** | Major city | 1 | reference | 0.39 | 1 | reference | 0.07 | 1 | reference | 0.19 |
|  | Regional/Remote | 0.85 | 0.58-1.21 |  | 1.30 | 0.97-1.72 |  | 0.80 | 0.57-1.10 |  |
| **Age group** | <55 | 1 | reference |  | 1 | reference |  | 1 | reference |  |
|  | 55-64 | 0.82 | 0.53-1.28 | 0.37 | **0.62** | 0.43-0.90 | 0.01 | 0.84 | 0.56-1.27 | 0.40 |
|  | 65-74 | 0.74 | 0.49-1.28 | 0.15 | **0.43** | 0.30-0.61 | <0.001 | 0.88 | 0.61-1.31 | 0.53 |
|  | 75-84 | **0.56** | 0.37-0.86 | 0.01 | **0.37** | 0.27-0.53 | <0.001 | 0.86 | 0.59-1.26 | 0.42 |
|  | ≥85 | **0.38** | 0.24-0.62 | <0.001 | **0.23** | 0.16-0.35 | <0.001 | **0.51** | 0.33-0.78 | 0.002 |
| **Episode Start Year** | - | **0.83** | 0.78-0.88 | <0.001 | **0.94** | 0.90-0.99 | 0.01 | **0.83** | 0.79-0.87 | <0.001 |
| **AKPS at start of episode** | - | **0.99** | 0.99-1.00 | 0.04 | **0.99** | 0.98-1.00 | 0.002 | **0.98** | 0.98-0.99 | <0.001 |
| **Service type** | Community | 1 | reference | 0.28 | 1 | reference | <0.001 | 1 | reference | 0.001 |
|  | In-patient | 0.88 | 0.70-1.11 |  | **1.49** | 1.24-1.80 |  | **0.72** | 0.59-0.87 |  |

*Abbreviations: CI, Confidence Interval; AKPS, Australia-modified Karnofsky Performance Status.*

**High psychological distress, pain and carer distress defined as a PCPSS of moderate or severe (i.e. low distress defined as a PCPSS of absent or mild)*

Table S4: Likelihood of patients with pancreatic cancer experiencing high symptom distress at first episode of specialist palliative care according to patient/service level characteristics

| **Patient/service level characteristics** | **Likelihood of experiencing high symptom distress at first episode of specialist PC** |
| --- | --- |
| **Female sex**  [Reference group: Male sex] | - ↓ likely to present with high family/carer problems. - No difference in pain, fatigue, appetite distress, or psychological/spiritual problems. |
| **Country of birth outside of Australia**  [Reference group: Australia] | - ↓ likely to present with high appetite distress or fatigue. - No difference in pain, psychological/spiritual problems or family/carer problems. |
| **Preferred language other than English**  [Reference group: English] | - ↓ likely to present with fatigue - ↑ likely to present with high family/carer problems. - No difference in pain, appetite distress, or psychological/spiritual problems. |
| **Regional/Remote location of residence**  [Reference group: Major city] | - No difference in pain, fatigue, appetite distress, psychological/spiritual or family/carer problems. |
| **Age ≥ 55 years**  [Reference group: <55] | - ↓ likely to present with high pain, psychological/spiritual or family/carer problems. - No difference in fatigue or appetite distress. |
| **Increasing performance status score**  [Reference group: Lower performance status score] | - ↓ likely to present with high pain, fatigue, psychological/spiritual or family/carer problems. - No difference in appetite distress. |
| **Recent year of first episode**  [Reference group: Earlier year of first episode] | - ↓ likely to present with high pain, fatigue, appetite distress, psychological/spiritual or family/carer problems. |
| **In-patient service setting**  [[Reference group: Community] | - ↑ likely to present with high pain. - ↓ likely to present with high fatigue, psychological/spiritual or family/carer problems. - No difference in appetite distress. |

Table S5: Characteristics of lower^ and higher survival cancer^?^ cases at first episode**^** of palliative care registered in the Palliative Care Outcomes Collaboration dataset between 2014-2020

|  | | **Initial presentation cohort^#^** | |
| --- | --- | --- | --- |
|  |  | **n (%)** | |
|  | | **Lower survival cancers** | **Higher survival cancers** |
| **Number of first episodes of PC** | | 12,934 | 20,013 |
| **Year of first episode** | **2014** | 1765 (13.6) | 2585 (12.9) |
|  | **2015** | 1691 (13.1) | 2357 (11.8) |
|  | **2016** | 1814 (14.0) | 2904 (14.5) |
|  | **2017** | 1837 (14.2) | 2941 (14.7) |
|  | **2018** | 1806 (14.0) | 2923 (14.6) |
|  | **2019** | 1911 (14.8) | 3004 (15.0) |
|  | **2020** | 2110 (16.3) | 3299 (16.5) |
| **Sex** | **Male** | 7534 (58.2) | 9923 (49.6) |
|  | **Female** | 5400 (41.8) | 10,090 (50.4) |
| **Country of Birth** | **Australia** | 6384 (49.4) | 10,376 (51.8) |
|  | **Other** | 6550 (50.6) | 9,637 (48.2) |
| **Preferred Language** | **English** | 10,794 (83.5) | 17,165 (85.8) |
|  | **Non-English** | 2140 (16.5) | 2848 (14.2) |
| **Location of residence** | **Major city** | 11393 (88.1) | 17,238 (86.1) |
|  | **Regional/Remote** | 1541 (11.9) | 2,775 (13.9) |
| **Age group** | **<55** | 1101 (8.5) | 2477 (12.4) |
|  | **55-64** | 2081 (16.1) | 3056 (15.3) |
|  | **65-74** | 3660 (28.3) | 4789 (23.9) |
|  | **75-84** | 3793 (29.3) | 5393 (26.9) |
|  | **85+** | 2278 (17.6) | 4221 (21.1) |
| **Phase type at first episode** | **Stable** | 4461 (34.5) | 6957 (34.8) |
|  | **Unstable** | 2184 (16.9) | 3356 (16.8) |
|  | **Deteriorating** | 5675 (43.9) | 8661 (43.3) |
|  | **Terminal** | 610 (4.7) | 1038 (5.2) |

*^#^Includes cases who were registered as having an episode identifier of ‘1’ and were referred to their first episode from a non-PC service.*

*^^^Lower survival cancers defined as having a 5-year survival rate less than 50% and include cancers of the central nervous system, lung and other gastrointestinal cancers*

*^?^Higher survival cancers defined as having a 5-year survival rate greater than or equal to 50% and include cancers of the bone and soft tissue, breast, colorectal, haematological, head and neck, prostate, other urological, gynaecological and skin.*

*^^^Episode refers to a continuous period of care for a patient in one setting (i.e. hospital, private residence, residential aged care facility, etc.). An episode begins the day the patient is assessed by the palliative care provider and there is agreement between the patient and the service that the patient is ready to receive palliative care.*

*Note: Numbers may not add to 100% due to missing data*

Table S6(i): Odds of high PCOC SAS* score in pancreatic cancer compared to higher survival cancers^ at initial presentation to palliative care

|  | **Pain** | | | **Fatigue** | | | **Appetite** | | |
| --- | --- | --- | --- | --- | --- | --- | --- | --- | --- |
|  | **OR** | **95% CI** | **P value** | **OR** | **95% CI** | **P value** | **OR** | **95% CI** | **P value** |
| **Higher survival cancers** | 1 | reference | 0.04 | 1 | reference | <0.001 | 1 | reference | <0.001 |
| **Pancreatic cancer** | **1.11** | 1.00-1.23 |  | **1.20** | 1.10-1.31 |  | **1.57** | 1.42-1.73 |  |

*Abbreviations: CI, Confidence Interval*

**High distress from pain, fatigue and appetite defined as a PCOC SAS pain score of ≥4 (i.e. low distress from pain, fatigue or appetite defined as a PCOC SAS pain score <4)*

*^^^Higher survival cancers defined as having a 5-year survival rate greater than or equal to 50% and include cancers of the bone and soft tissue, breast, colorectal, haematological, head and neck, prostate, other urological, gynaecological and skin.*

Table S6(ii): Odds of high PCPSS score* in pancreatic cancer compared to higher survival cancers^ at initial presentation to palliative care

|  | **Pain** | | | **Psychological/Spiritual Problems** | | | **Family/Carer Problems** | | |
| --- | --- | --- | --- | --- | --- | --- | --- | --- | --- |
|  | **OR** | **95% CI** | **P value** | **OR** | **95% CI** | **P value** | **OR** | **95% CI** | **P value** |
| **Higher survival cancers** | 1 | reference | 0.25 | 1 | reference | 0.42 | 1 | reference | 0.57 |
| **Pancreatic cancer** | 1.06 | 0.96-1.17 |  | 0.95 | 0.85-1.07 |  | 1.03 | 0.93-1.14 |  |

*Abbreviations: CI, Confidence Interval*

**High psychological distress, pain and carer distress defined as a PCPSS of moderate or severe (i.e. low distress defined as a PCPSS of absent or mild)*

*^^^Higher survival cancers defined as having a 5-year survival rate greater than or equal to 50% and include cancers of the bone and soft tissue, breast, colorectal, haematological, head and neck, prostate, other urological, gynaecological and skin.*

Table S7(i): Odds of high PCOC SAS score* in pancreatic cancer compared to lower survival cancers^^^ at initial presentation to palliative care

|  | **Pain** | | | **Fatigue** | | | **Appetite** | | |
| --- | --- | --- | --- | --- | --- | --- | --- | --- | --- |
|  | **OR** | **95% CI** | **P value** | **OR** | **95% CI** | **P value** | **OR** | **95% CI** | **P value** |
| **Lower survival cancers** | 1 | reference | <0.001 | 1 | reference | <0.001 | 1 | reference | <0.001 |
| **Pancreatic cancer** | **1.27** | 1.15-1.41 |  | **1.19** | 1.09-1.30 |  | **1.47** | 1.33-1.63 |  |

*Abbreviations: CI, Confidence Interval*

**High distress from pain, fatigue and appetite defined as a PCOC SAS pain score of ≥4 (i.e. low distress from pain, fatigue or appetite defined as a PCOC SAS pain score <4)*

*^^^Lower survival cancers defined as having a 5-year survival rate less than 50% and* *include cancers of the central nervous system, lung and other gastrointestinal cancers.*

Table S7(ii): Odds of high PCPSS score* in pancreatic cancer compared to lower survival cancers*^^^* at initial presentation to palliative care

|  | **Pain** | | | **Psychological/Spiritual Problems** | | | **Family/Carer Problems** | | |
| --- | --- | --- | --- | --- | --- | --- | --- | --- | --- |
|  | **OR** | **95% CI** | **P value** | **OR** | **95% CI** | **P value** | **OR** | **95% CI** | **P value** |
| **Lower survival cancers** | 1 | reference | <0.001 | 1 | reference | 0.58 | 1 | reference |  |
| **Pancreatic cancer** | **1.21** | 1.09-1.34 |  | 0.97 | 0.86-1.09 |  | 0.97 | 0.87-1.07 | 0.51 |

*Abbreviations: CI, Confidence Interval*

**High psychological distress, pain and carer distress defined as a PCPSS of moderate or severe (i.e. low distress defined as a PCPSS of absent or mild)*

*^^^Lower survival cancers defined as having a 5-year survival rate less than 50% and include cancers of the central nervous system, lung and other gastrointestinal cancers.*
